# Fatigue in adults with traumatic brain injury: predictors and consequences. A systematic review of longitudinal study protocols

**DOI:** 10.1186/2046-4053-2-57

**Published:** 2013-07-11

**Authors:** Tatyana Mollayeva, Tetyana Kendzerska, Shirin Mollayeva, Colin M Shapiro, Angela Colantonio, J David Cassidy

**Affiliations:** 1 Graduate Department of Rehabilitation Science, Faculty of Medicine, University of Toronto, Toronto, Canada; 2 Collaborative Program in Neuroscience, University of Toronto, Toronto, Canada; 3 Toronto Rehabilitation Institute, University of Toronto, 550 University Avenue, Rm 11207, Toronto, Ontario M5G 2A2, Canada; 4 Institute of Health Policy, Management and Evaluation, University of Toronto, 155 College Street, Suite 425, Toronto, Ontario M5T 3M6, Canada; 5 Faculty of Arts and Science, University of Toronto, Toronto, Canada; 6 Acquired Brain Injury Research Lab, University of Toronto, 160-500 University Ave, Toronto, Ontario M5G 1V7, Canada; 7 Toronto Western Hospital, University Health Network, 399 Bathurst Street, Rm 7MP421, Toronto, Ontario M5T 2S8, Canada; 8 Youthdale Child & Adolescent Sleep Clinic, Toronto, Ontario, Canada; 9 Department of Occupational Science and Occupational Therapy, University of Toronto, Toronto, Ontario, Canada; 10 Institute of Sport Science and Clinical Biomechanics, Faculty of Health, University of Southern Denmark, Campusvej 55, DK-5230, Odense M, Denmark; 11 Division of Health Care and Outcomes Research, Toronto Western Research Institute, Toronto, Canada; 12 Saunderson Family Chair in Acquired Brain Injury Research, Toronto Rehabilitation Institute, University of Toronto, 160-500 University Ave, Toronto, Ontario M5G 1V7, Canada

**Keywords:** Post-traumatic fatigue, Traumatic brain injury, Rehabilitation, Protocol, Systematic review

## Abstract

**Background:**

Despite strong indications that fatigue is the most common and debilitating symptom after traumatic brain injury, little is known about its frequency, natural history, or relation to other factors. The current protocol outlines a strategy for a systematic review that will identify, assess, and critically appraise studies that assessed predictors for fatigue and the consequences of fatigue on at least two separate time points following traumatic brain injury.

**Methods/design:**

MEDLINE, EMBASE, the Cochrane Database of Systematic Reviews, CINAHL, and PsycINFO will be systematically searched for relevant peer-reviewed studies. Reference lists of eligible papers will also be searched. All English language studies with a longitudinal design that focus on fatigue in adults with primary-impact traumatic brain injury will be included. Studies on fatigue following brain injury due to secondary pathological processes (intracranial complications, edema, ischemia/infarction, and systemic intracranial conditions) will be excluded. Excluded studies, along with the reasons for exclusion will be reported. Two independent reviewers will conduct all levels of screening, data abstraction, and quality appraisal. Randomized control trial data will be treated as a cohort. The quality will be assessed using the criteria defined by Hayden and colleagues. The review will be conducted and reported in compliance with the Preferred Reporting Items for Systematic Reviews and Meta-Analyses guidelines.

**Conclusions:**

The review will summarize the current knowledge in the field with the aim of increasing understanding and guiding future research on the associations between fatigue and clinically important factors, as well as the consequences of fatigue in traumatic brain injury. PROSPERO registry number: CRD42013004262.

## Background

Traumatic brain injury (TBI), defined as ‘an alteration in brain function, or other evidence of brain pathology, caused by an external force,’ [1] is a major global health problem. According to the World Health Organization, TBI is predicted to surpass many diseases as a major cause of death and disability by the year 2020 [2]. The incidence of TBI is highest among young people [3]; any adverse long-term effects will impact the person’s ability to return to their previous social roles, including pre-disability employment. Fatigue is commonly reported to be one of the most disabling symptoms in patients following TBI [4], and occurs in 21% to 73% of affected individuals. Fatigue is a symptom, rather than a diagnosis, which is extremely difficult to clarify and operationalize. A generally accepted universal definition of fatigue does not exist, nor is there any conceptual framework for studying fatigue in TBI. Nevertheless, discussions in the literature make a distinction between central fatigue (due to the dysfunction of supratentorial structures involved in mentation) and peripheral fatigue (of physical, metabolic, or muscular origin) [5]. The number of studies including post-TBI fatigue as an outcome measure has rapidly increased over the past decade. Fatigue is reported to be significantly higher in persons who have sustained TBI than in those who have not [6-8]. Higher levels of post-TBI fatigue have been reported to lead to a poorer quality of life [8].

There are several published narrative reviews of fatigue after TBI [9-11]. Systematic reviews are scientifically more robust than narrative reviews and are therefore a more valid source of information and less prone to bias [12]. Thus, our current aim was to identify, appraise, and synthesize all available longitudinal studies on post-traumatic fatigue in an attempt to (1) determine prognostic factors for fatigue onset in patients with TBI; (2) determine the course of fatigue in patients with TBI; and (3) determine the health consequences of fatigue in patients with TBI. The current protocol outlines a strategy for this systematic review.

## Methods/design

The review will be conducted and reported in compliance with the Preferred Reporting Items for Systematic Reviews and Meta-Analyses (PRISMA) guidelines [13]. In accordance with these guidelines, our systematic review protocol was registered with the International Prospective Register of Systematic Reviews (PROSPERO) [14] on 25 April 2013 (registration number CRD42013004262).

### Search methods for the identification of studies

MEDLINE, CINAHL, and PsycINFO were systematically searched from 1946, 1980, and 1806, respectively; EMBASE was searched from 1974, and the Cochrane Database of Systematic Reviews from 2005 to early April 2013. The complete search strategy can be found in Additional file 1.

Search terms for fatigue were developed by reviewing the literature found in a previous systematic review of fatigue [15,16], and by consulting an information specialist at the Toronto Rehabilitation Institute. Three categories of search terms will be used (Table 1). We will limit our search to studies in English on adults aged 18 years and over (with no upper age limit). We will include all articles with the exception of papers assessing fatigue due to secondary pathological processes after brain injury (for example, edema, intracranial hemorrhages, ischemia/infarction, and systemic intracranial conditions).

**Table 1:** Terms used in search

**Brain injuries**	**Fatigue**	**Models**
- exp Brain Injuries/	- fatigue/or fatigue syndrome, chronic/or asthenia/or mental fatigue/or muscle fatigue/or lethargy/listlessness or letarg$ or apath$ or malaise).tw	- (‘Models, Theoretical’[Mesh] OR ‘Causality’[Mesh] OR ‘Etiology’[Subheading])
	- ((low or lack) adj5 energy).tw	
- craniocerebral trauma/or exp brain injuries/or coma, post-head injury/or head injuries, closed/or head injuries, penetrating/or exp intracranial hemorrhage, traumatic/or exp skull fractures/		

In addition to the electronic databases, a manual search of the reference lists of reviews from relevant journals published between 1990 and 2013 (for example, the *Journal of Head Trauma Rehabilitation and Neurorehabilitation*) will be conducted to ensure our search is complete.

### Criteria for considering studies for this review

We will include peer-reviewed published longitudinal studies (that is, studies that have assessed fatigue on at least two separate occasions) in English, conducted with adults who have a clinical diagnosis of TBI. Any measures of fatigue will be accepted (for example, the presence or absence of fatigue determined using a single question, a case definition on a fatigue scale, or fatigue scores reported as a continuous variable). For the purpose of this review, we will define human fatigue as the undesirable state produced by effort – the physical or mental effort of doing work [17]. We will also include studies that were primarily designed to investigate the predictors and consequences of fatigue in the TBI population.

The participants in the studies must be men or women aged 18 years or older, with TBI defined by clinical criteria. For the purpose of this review, we will focus on TBI cases of primary-impact injury only. The operational definitions for the clinical identification of primary-impact TBI include: (i) concussion: ‘a trauma-induced alteration in mental status that may or may not involve loss of consciousness’ [18]; (ii) coup and contrecoup (damage at the site or opposite to the site of a blow); (iii) contusion (hemorrhagic); and (iv) diffuse axonal injury. There is no restriction on how the fatigue was diagnosed or assessed.

### Exclusion criteria

We will not include studies that focus on a different but parallel topic to fatigue (for example, sleepiness, impaired alertness, or vigilance). The cause of a patient’s symptoms can be hard to determine on a single item scale as patients with TBI may use vague words to describe their state (for example, sleepiness vs. fatigue). Sleepiness is a basic physiological state, the presence and intensity of which can be inferred by how readily sleep onset occurs, how easily sleep is disrupted, and how long sleep endures [18]. The utilized measures, and items within them, will be used to distinguish fatigue from sleepiness. For example, symptoms of sleepiness are unintended episodes of falling asleep during the daytime or elevated numbers on standardized sleepiness scales; symptoms of fatigue are muscular weakness or lack or energy. We recognize that some fatigue scales may include items for sleepiness. As part of our review, we will report whether utilized fatigue scales were uni- or multi-dimensional. In addition, a detailed review of the medications (whenever available) will be undertaken and reported, as many medications, including those commonly used in the TBI population (for example, antiepileptics, antipsychotics, antihistamines, corticosteroids, and antidepressants), can cause fatigue [19].

We will also not include studies about fatigue after brain injury due to secondary pathological processes (for example, edema, intracranial hemorrhages, ischemia/infarction, and systemic intracranial conditions), and also letters to editors, reviews without data, case reports, conference abstracts and unpublished manuscripts.

### Types of study design

#### Criteria for considering studies for this review

Both experimental studies (intervention and effectiveness studies with a prospective longitudinal design) and non-experimental studies (observational studies, that is, cohort and case control studies) will be considered for this review.

#### Selection of studies

All hits will be saved in EndNote and duplicates removed. For the first level of screening, two reviewers (TM and TK) will read the titles and abstracts of all the citations from the electronic database searches and remove all citations not related to primary-impact TBI. For the second level of screening, if the title or abstract suggests that the study might meet the inclusion criteria then each reviewer will individually assess the full article; any conflicting views will be resolved by consultation between the reviewers, or by seeking advice from other experts (AC, CMS, and JDC). Studies failing to meet the inclusion criteria will be excluded, and the reasons listed in the table of the characteristics of excluded studies (Additional file 2).

#### Data extraction

For studies fulfilling the inclusion criteria, the two independent reviewers (TM and TK) will independently extract data into data collection forms grouped according to their design. Data from observational studies will be used to address all three research objectives. Randomized control trial (RCT) studies will be treated as cohorts: we will utilize the control data (for example, the untreated group) from RCT studies to address the second research objective (to determine the course of fatigue) in patients with TBI.

Data from RCTs will be used to answer the research question on the course of fatigue in TBI only. We will use the data to: (1) identify RCTs with a focus on fatigue as a primary outcome; (2) identify treatment arms (for example, intervention/no intervention); (3) report on the course of fatigue in untreated arms (for example, no intervention arms); and (4) report on the course of fatigue for both arms where treatment did not produce an effect.

The abstracted data will include: (1) study characteristics (author names, publication year, country of study, study setting, study design, sample size, methods of measuring fatigue and other variables such as factors, number of participants assessed for fatigue at each time point, time between assessments, and time since injury for each follow-up); (2) participant characteristics (mean age, sex, definition of TBI, localization of injury, and injury severity); (3) a detailed review of medication; and (4) results (reported frequencies of fatigue and other factors, and reported associations between fatigue and other variables) (Tables 2 and 3).

**Table 2:** Data from studies assessing fatigue at two or more time points after traumatic brain injury

**Abstracted data**	
First author, date, country, study setting	
Sample size (*N*), injury severity and definition (% of total), time of assessment post TBI and number of participants that completed assessment at each time point, mean age (or age ± SD), sex (*M*,%)	
Study design, follow-up time	
Main statistical method	
Measure of fatigue	
Medication regime (if reported)	
Results	Frequency of fatigue at each time point (95% CI)
	Differences in fatigue scores across time points (if reported)

TBI, traumatic brain injury.

**Table 3:** **Data from longitudinal studies reporting associations between fatigue and other variables after traumatic brain injury**^**a**^

**Abstracted data**	
First author, date, country, study setting	
Sample size (*N*), injury severity and definition (% of total), time since injury (TSI), mean age (or age ± SD), sex (*M*,%)	
Study design, follow-up time	
Main statistical method	
Measure of fatigue	
Medication regime (if reported)	
Results	Associations between fatigue and other factors post-TBI*
	Measure of other factor(s)

^a^ Only results from multivariable analysis will be reported. Univariate associations will be reported only if an adjustment was not performed. Where possible, 95% confidence intervals will be reported.

TBI, traumatic brain injury.

### Analysis

#### Data synthesis

We will divide the results of each study into three main categories: the course of fatigue, prognostic factors, and the consequences of fatigue. To determine the course of fatigue per study an overall percentage of fatigue as a primary outcome measure will be reported. Fatigue resolution, exacerbation, and no change will be identified and reported as: less fatigue, more fatigue, no fatigue, and so on. Next, a range and median (using the overall percentages) will be calculated for the number of studies reporting on the outcome measure.

Prognostic factors associated with fatigue will be extracted from all cohorts (and from untreated and without an effect groups in RCTs). All factors influencing the course of fatigue as reported by the author will be considered to be prognostic factors. We will consider a prognostic association as significant if (1) the reported *P* value is less than 0.05; (2) the author reported that an association was significant; (3) the 95% confidence intervals around a rate ratio or similar statistic did not include 1. Where a prognostic factor was assessed with respect to the outcome at a number of time points in one cohort, data will be extracted and reported for each follow-up.

To address our third research objective (the health consequences of fatigue in TBI) we will evaluate the literature regarding putative negative effects (for example, quality of life, morbidity, mortality, costs, and satisfaction) associated with fatigue after TBI at each time point. The instrument that was used (for example, single item, standardized/non-standardized, and validated/non-validated) for reporting will be defined and specified. Since establishing causal links between fatigue and health outcomes may be difficult in TBI, we will use criteria such as the following in our report: temporal relation, lack of alternative causes, outcome response to alleviation or exacerbation of fatigue over time. Confounding factors (such as sociodemographic characteristics, severity of injury, and comorbidities) that may affect the generalizability of the study and interpretation of results will be explored and clearly described. In addition, we will report on the possible effects of individual study quality indicators (for example, the follow-up period and instrument used to measure the outcome), on study design and study size [12].

#### Methodological quality and risk of bias assessment

Study quality will be assessed independently by two reviewers (TK and TM) using the guidelines developed by Hayden *et al*. for assessing prognostic studies [20]. The appraisal will consist of two steps. The first step will assess the items related to six potential sources of bias (study participation, attrition, prognostic factors, outcome measurements, confounding measurements and account, and analyses). The second step will classify potential biases as ‘Yes,’ ‘Partly,’ ‘No,’ or ‘Unsure.’ Then the reviewers will decide if a study has a fatal flaw or bias. If so, that study will be categorized as having a ‘high risk of bias.’ To ensure that the bias assessment is explicit, we will record in the supplements those aspects of the trial methods used to make this judgment and the judgment itself, including the trial method on which the decision to exclude was based (Additional file 2). For the studies with a ‘low risk of bias’, we will abstract data on the relations between fatigue and other variables. Any statistical measure of association (for example, odds ratio, hazard ratio, or relative risk) will be reported and laid out as Table 4.

**Table 4:** **The most common significant predictors of post-traumatic fatigue**^**a**^

						
**Study, author and year**	**Factor 1**	**Factor 2**	**Factor 3**	**Factor 4**	**Factor 5**	**Factor**

a * No correlation reported in fully adjusted model between fatigue post-TBI and the factor. · Positive significant association. 

Negative significant association. ○ No significant association reported in multivariable model. ‘―’ Factor was not included in the model.

To summarize the level of evidence, we will utilize a system similar to the Scottish Intercollegiate Guidelines Network Methodology [21]: (i) ‘++’ when all, or most of the quality criteria proposed by Hayden *et al.*[20] are fulfilled (with at most one ‘Partly’ in the potential sources of bias); (ii) ‘+’ when some of the criteria are fulfilled; and (iii) ‘-’ when few or none of the criteria are fulfilled (at least one ‘Yes’). We will refer to group (i) as ‘high-quality studies’ and group (ii) as ‘moderate-quality studies’. This summary will be laid as Table 5.

**Table 5:** **Quality assessment of studies using guidelines developed by Hayden *et al*.**[20]

							
**Study**	**Study participation**	**Study attrition**	**Prognostic factor**	**Outcome**	**Confounding measurement and account**	**Analysis**	**Overall assessment of the study**

Given the diversity of definitions for fatigue, populations of interest, and the statistical methodologies used to express association, a meta-analysis will be not conducted. The findings from studies with sufficient quality will be synthesized by means of tabulation and qualitative description. Only brief summary results will be provided for studies that do not meet our quality criteria.

#### Dealing with missing data

The primary authors will be contacted if there is missing data. Where possible, the proportion of missing data will be stated, along with possible reasons. If there are duplicate publications and companion papers of a primary study, we will try to maximize the yield of information by a simultaneous evaluation of all available data. If there is any doubt, the original publication (usually the oldest) will take priority.

## Discussion

To the best of our knowledge, this is the first systematic review of fatigue in TBI, and has the potential to significantly improve methodological understanding of its frequency, natural history, and associated factors. Our systematic review has a number of strengths. First, we will use extensive search strategies, making it unlikely that we will miss relevant studies. Second, our strategies are intentionally sensitive, rather than specific. Third, two authors will extract the data independently, thus reducing the chance of errors occurring in data extraction. Furthermore, we will use a rigorously developed protocol, with clearly defined inclusion and exclusion criteria, decided in advance of performing the searches. Finally, expanded synonyms of the word ‘fatigue’ and ‘brain injuries’ will be used for the search.

A potential weakness of this review is that we have specified that only studies published in English will be included, and therefore, any relevant studies published in other languages will be omitted. An attempt will be made to identify non-English language papers (for example, titles and abstracts are usually translated into English in many databases) and document their existence and the reason for exclusion will be recorded as ‘language’. In addition, all of the articles to be included in this review will have been peer reviewed and, as such, there is some publication bias.

### Dissemination plans

An extensive knowledge translation strategy will be implemented at the conclusion of this review. The target audience is rehabilitation practitioners and other professionals (for example, TBI specialists, sleep specialists, and psychiatrists) who work with the TBI population. The results of this systematic review will be presented at relevant meetings: locally (for example, the Rehabilitation Research Day and the Southern Ontario Neuroscience Association Annual Meeting), nationally (for example, the ABI Canadian Conference and the Canadian Sleep Society National Meeting), and internationally (the Brain Injury World Congress and the Sleep Society World Conference). The results will be published in a peer-reviewed journal for the appropriate academic and clinical audience.

### Implications

Fatigue is a common post-TBI symptom, and is likely to be long lasting. This systematic review and best-evidence synthesis has been planned to inform clinical practice and future research by formulating new questions, and encouraging scientists and clinicians to examine certain relations in greater detail, and to discover how disease-specific processes can contribute to fatigue in TBI.

## Abbreviations

PRISMA: Preferred Reporting Items for Systematic Reviews and Meta-Analyses; RCT: randomized control trial; TBI: traumatic brain injury.

## Competing interests

The authors have no conflicts of interest to declare pertaining to this review.

## Authors’ contributions

TM, TK, SM, CMS, JDC and AC contributed to the conception and design of the review. TM developed the idea and designed the protocol. TK co-developed the idea and supported TM in the development of the search strategy. SM was responsible for building the graphic data representation and helped to draft the proposal. AC provided expertise at each level and also reviewed the protocol. CMS and JDC inspired the idea and critically reviewed the protocol. TM wrote the first draft, which was revised by TK, AC, CMS, and JDC. TM and TK registered the protocol. All authors read and approve the final manuscript.

## Supplementary Material

Additional file 1 **Search strategies.** This file provides the list of search terms used to search MEDLINE, EMBASE, Cochrane, CINAHL and PsycINFO. Click here for file

Additional file 2 **Characteristics of excluded studies.** This file has a table of excluded studies. Click here for file
