# Supplementary material for: Fatigue in adults with traumatic brain injury: predictors and consequences. A systematic review of longitudinal study protocols
Source: Syst Rev. 2013 Jul 11;2:57. doi: 10.1186/2046-4053-2-57 (PMC3717139; doi:10.1186/2046-4053-2-57)
Supplement: Additional file 1 — Search strategies. This file provides the list of search terms used to search MEDLINE, EMBASE, Cochrane, CINAHL and PsycINFO. [file 2046-4053-2-57-S1.docx]

**Additional file 1. Search strategy**

**Medline: 724 (run 2013.05.16)**

Database: Ovid MEDLINE(R) <1946 to April Week 3 2013>

Search Strategy:

--------------------------------------------------------------------------------

1 [CONCEPT 1 - TRAUMATIC BRAIN INJURY] (0)

2 exp brain injuries/ (46302)

3 Craniocerebral Trauma/ (18396)

4 exp Head Injuries, Closed/ (6701)

5 exp Skull Fractures/ (17434)

6 mTBI*2.tw. (615)

7 tbi*2.tw. (12141)

8 concuss*.tw. (3146)

9 ((head* or cerebr* or crani* or capitis* or brain* or forebrain* or skull* or hemispher* or intracran* or orbit*) adj2 (injur* or trauma* or lesion* or damag* or wound* or destruction* or swell* or oedema* or edema* or fracture* or contusion* or commotion* or pressur*)).tw. (124592)

10 ((brain* or cerebr* or intracerebr* or crani* or intracran* or head* or subarachnoid* or subdural* or epidural* or extradural*) adj (haematoma* or hematoma* or hemorrhag* or haemorrhag* or pressur* or bleed*)).tw. (54472)

11 or/2-10 (195833)

12 [CONCEPT 2 - FATIGUE] (0)

13 exp Fatigue/ (18828)

14 Asthenia/ (1353)

15 Lethargy/ (168)

16 Fatigue Syndrome, Chronic/ (4242)

17 fatigue*.tw. (50301)

18 astheni*.tw. (3667)

19 tiredness.tw. (2352)

20 tire.tw. (669)

21 tired.tw. (1183)

22 weary.tw. (115)

23 letharg*.tw. (4886)

24 apath*.tw. (3540)

25 malaise*.tw. (4732)

26 Neurasthenia/ (1282)

27 neurastheni*.tw. (666)

28 ((low or lack) adj5 energy).tw. (15279)

29 Neurocirculatory Asthenia/ (1278)

30 or/13-29 (94303)

31 11 and 30 [Concept 1 AND 2] (1290)

32 [CONCEPT 5 - ETIOLOGY?] (0)

33 model*.tw. (1434517)

34 theor*.tw. (283744)

35 framework*.tw. (103130)

36 predict*.tw. (782231)

37 etiology*.tw. (115359)

38 pathophysiology.tw. (78342)

39 factor*.tw. (1941388)

40 pp.fs. (1336570)

41 et.fs. (1964925)

42 risk*.mp. (1425456)

43 exp Cohort Studies/ (1262672)

44 between group*.tw. (60317)

45 cohort*.tw. (225420)

46 or/33-45 (7325798)

47 31 and 46 (1008)

48 limit 47 to english language (789)

49 (exp child/ or exp infant/) not ((exp child/ or exp infant/) and (exp adolescent/ or exp aged/ or exp adult/)) (999415)

50 48 not 49 (724)

***************************

**Embase : 1128 (run 2013.04.12)**

Database: Embase <1974 to 2013 April 11>

Search Strategy:

--------------------------------------------------------------------------------

1 [CONCEPT 1 - TRAUMATIC BRAIN INJURY] (0)

2 exp brain injury/ (112090)

3 head injury/ (38180)

4 [exp Head Injuries, Closed/] (0)

5 [exp Skull Fractures/] (0)

6 mTBI*2.tw. (1061)

7 tbi*2.tw. (18793)

8 concuss*.tw. (4165)

9 ((head* or cerebr* or crani* or capitis* or brain* or forebrain* or skull* or hemispher* or intracran* or orbit*) adj2 (injur* or trauma* or lesion* or damag* or wound* or destruction* or swell* or oedema* or edema* or fracture* or contusion* or commotion* or pressur*)).tw. (169808)

10 ((brain* or cerebr* or intracerebr* or crani* or intracran* or head* or subarachnoid* or subdural* or epidural* or extradural*) adj (haematoma* or hematoma* or hemorrhag* or haemorrhag* or pressur* or bleed*)).tw. (75567)

11 or/2-10 (282349)

12 [CONCEPT 2 - FATIGUE] (0)

13 Fatigue/ (96726)

14 Asthenia/ (17226)

15 Lethargy/ (10278)

16 Chronic Fatigue Syndrome/ (7081)

17 fatigue*.tw. (73333)

18 astheni*.tw. (5903)

19 tiredness.tw. (3783)

20 tire.tw. (1381)

21 tired.tw. (1746)

22 weary.tw. (160)

23 letharg*.tw. (6577)

24 apath*.tw. (5227)

25 malaise*.tw. (6850)

26 Neurasthenia/ (1694)

27 neurastheni*.tw. (901)

28 ((low or lack) adj5 energy).tw. (20790)

29 lassitude/ (563)

30 exaustion/ (0)

31 muscle fatigue/ (8547)

32 or/13-31 (198106)

33 11 and 32 [Concept 1 AND 2] (3160)

34 [CONCEPT 3 - ADULT] (0)

35 limit 33 to "all adult (19 plus years)" [Limit not valid in Embase; records were retained] (3160)

36 [CONCEPT 4 - ENGLISH] (0)

37 limit 35 to english language (2667)

38 [CONCEPT 5 - ETIOLOGY?] (0)

39 model*.tw. (1864733)

40 theor*.tw. (377339)

41 framework*.tw. (133107)

42 predict*.tw. (1044573)

43 [or/39-51] (0)

44 [CONCEPT 1 - TRAUMATIC BRAIN INJURY] (0)

45 exp brain injury/ (112090)

46 head injury/ (38180)

47 [exp Head Injuries, Closed/] (0)

48 [exp Skull Fractures/] (0)

49 mTBI*2.tw. (1061)

50 tbi*2.tw. (18793)

51 concuss*.tw. (4165)

52 ((head* or cerebr* or crani* or capitis* or brain* or forebrain* or skull* or hemispher* or intracran* or orbit*) adj2 (injur* or trauma* or lesion* or damag* or wound* or destruction* or swell* or oedema* or edema* or fracture* or contusion* or commotion* or pressur*)).tw. (169808)

53 ((brain* or cerebr* or intracerebr* or crani* or intracran* or head* or subarachnoid* or subdural* or epidural* or extradural*) adj (haematoma* or hematoma* or hemorrhag* or haemorrhag* or pressur* or bleed*)).tw. (75567)

54 or/45-53 (282349)

55 [CONCEPT 2 - FATIGUE] (0)

56 Fatigue/ (96726)

57 Asthenia/ (17226)

58 Lethargy/ (10278)

59 Chronic Fatigue Syndrome/ (7081)

60 fatigue*.tw. (73333)

61 astheni*.tw. (5903)

62 tiredness.tw. (3783)

63 tire.tw. (1381)

64 tired.tw. (1746)

65 weary.tw. (160)

66 letharg*.tw. (6577)

67 apath*.tw. (5227)

68 malaise*.tw. (6850)

69 Neurasthenia/ (1694)

70 neurastheni*.tw. (901)

71 ((low or lack) adj5 energy).tw. (20790)

72 lassitude/ (563)

73 exaustion/ (0)

74 muscle fatigue/ (8547)

75 or/56-74 (198106)

76 54 and 75 [Concept 1 AND 2] (3160)

77 [CONCEPT 4 - ENGLISH] (0)

78 [CONCEPT 5 - ETIOLOGY?] (0)

79 model*.tw. (1864733)

80 theor*.tw. (377339)

81 framework*.tw. (133107)

82 predict*.tw. (1044573)

83 etiology*.tw. (161459)

84 pathophysiology.tw. (107758)

85 factor*.tw. (2510163)

86 pp.fs. (0)

87 et.fs. (2146877)

88 risk*.mp. (2044856)

89 exp Cohort analysis/ (144187)

90 between group*.tw. (87662)

91 cohort*.tw. (330581)

92 or/79-91 (7772452)

93 76 and 92 (1594)

94 limit 93 to english language (1341)

95 limit 94 to (embryo <first trimester> or infant <to one year> or child <unspecified age> or preschool child <1 to 6 years> or school child <7 to 12 years> or adolescent <13 to 17 years>) (213)

96 94 not 95 (1128)

***************************

**Psychinfo: 453 (run 2013.04.18)**

Database: PsycINFO <1806 to April Week 2 2013>

Search Strategy:

--------------------------------------------------------------------------------

1 [CONCEPT 1 - TRAUMATIC BRAIN INJURY] (0)

2 exp traumatic brain injury/ (9867)

3 exp head injuries/ (4478)

4 [exp Head Injuries, Closed/] (0)

5 [exp Skull Fractures/] (0)

6 mTBI*2.tw. (595)

7 tbi*2.tw. (5334)

8 concuss*.tw. (1174)

9 ((head* or cerebr* or crani* or capitis* or brain* or forebrain* or skull* or hemispher* or intracran* or orbit*) adj2 (injur* or trauma* or lesion* or damag* or wound* or destruction* or swell* or oedema* or edema* or fracture* or contusion* or commotion* or pressur*)).tw. (40326)

10 ((brain* or cerebr* or intracerebr* or crani* or intracran* or head* or subarachnoid* or subdural* or epidural* or extradural*) adj (haematoma* or hematoma* or hemorrhag* or haemorrhag* or pressur* or bleed*)).tw. (3054)

11 or/2-10 (42925)

12 [CONCEPT 2 - FATIGUE] (0)

13 Fatigue/ (5551)

14 exp Asthenia/ (158)

15 sleepiness/ (1076)

16 Chronic Fatigue Syndrome/ (1447)

17 fatigue*.tw. (16491)

18 astheni*.tw. (763)

19 tiredness.tw. (922)

20 tire.tw. (160)

21 tired.tw. (801)

22 weary.tw. (148)

23 letharg*.tw. (899)

24 apath*.tw. (3162)

25 malaise*.tw. (919)

26 Neurasthenia/ (262)

27 neurastheni*.tw. (1060)

28 ((low or lack) adj5 energy).tw. (1049)

29 lassitude/ (0)

30 exaustion/ (0)

31 muscle fatigue/ (0)

32 or/13-31 (26897)

33 11 and 32 [Concept 1 AND 2] (611)

34 limit 33 to (childhood <birth to 12 years> or adolescence <13 to 17 years>) (90)

35 33 not 34 (521)

36 limit 35 to english language (453)

**************************

**Cochrane: 175 (run 2013.04.18)**

Database: EBM Reviews - Cochrane Database of Systematic Reviews <2005 to March 2013>

Search Strategy:

--------------------------------------------------------------------------------

1 [CONCEPT 1 - TRAUMATIC BRAIN INJURY] (0)

2 [exp brain injuries/] (0)

3 [Craniocerebral Trauma/] (0)

4 [exp Head Injuries, Closed/] (0)

5 [exp Skull Fractures/] (0)

6 mTBI*2.tw. (1)

7 tbi*2.tw. (56)

8 concuss*.tw. (30)

9 ((head* or cerebr* or crani* or capitis* or brain* or forebrain* or skull* or hemispher* or intracran* or orbit*) adj2 (injur* or trauma* or lesion* or damag* or wound* or destruction* or swell* or oedema* or edema* or fracture* or contusion* or commotion* or pressur*)).tw. (745)

10 ((brain* or cerebr* or intracerebr* or crani* or intracran* or head* or subarachnoid* or subdural* or epidural* or extradural*) adj (haematoma* or hematoma* or hemorrhag* or haemorrhag* or pressur* or bleed*)).tw. (465)

11 or/2-10 (968)

12 [CONCEPT 2 - FATIGUE] (0)

13 [exp Fatigue/] (0)

14 [Asthenia/] (0)

15 [Lethargy/] (0)

16 [Fatigue Syndrome, Chronic/] (0)

17 fatigue*.tw. (907)

18 astheni*.tw. (116)

19 tiredness.tw. (152)

20 tire.tw. (6)

21 tired.tw. (52)

22 weary.tw. (7)

23 letharg*.tw. (145)

24 apath*.tw. (130)

25 malaise*.tw. (173)

26 [Neurasthenia/] (0)

27 neurastheni*.tw. (15)

28 ((low or lack) adj5 energy).tw. (104)

29 [Neurocirculatory Asthenia/] (0)

30 or/13-29 (1378)

31 11 and 30 [Concept 1 AND 2] (175)

32 [CONCEPT 3 - ADULT] (0)

33 limit 31 to "all adult (19 plus years)" [Limit not valid; records were retained] (175)

34 [CONCEPT 4 - ENGLISH] (0)

35 limit 33 to english language [Limit not valid; records were retained] (175)

36 [CONCEPT 5 - ETIOLOGY?] (0)

37 model*.tw. (6293)

38 theor*.tw. (1656)

39 framework*.tw. (422)

40 predict*.tw. (2143)

41 etiology*.tw. (835)

42 pathophysiology.tw. (428)

43 factor*.tw. (5612)

44 [pp.fs.] (0)

45 [et.fs.] (0)

46 risk*.mp. (7477)

47 [exp Cohort Studies/] (0)

48 between group*.tw. (8061)

49 cohort*.tw. (1588)

50 or/37-49 (8087)

51 35 and 50 (175)

***************************

**Cinahl: 265 (run 2013.04.18)**

| Search ID# | Search Terms | Search Options | Last Run Via | Results |
| --- | --- | --- | --- | --- |
| S16 | (s7 and s14) not s15 | Search modes - Boolean/Phrase | Interface - EBSCOhost  Search Screen - Advanced Search  Database - CINAHL | 265 |
| S15 | S7 AND S14 | Limiters - Age Groups: All Infant, All Child  Search modes - Boolean/Phrase | Interface - EBSCOhost  Search Screen - Advanced Search  Database - CINAHL | 75 |
| S14 | S8 OR S9 OR S10 OR S11 OR S12 OR S13 | Search modes - Boolean/Phrase | Interface - EBSCOhost  Search Screen - Advanced Search  Database - CINAHL | 19,568 |
| S13 | (MH "Muscle Fatigue") | Search modes - Boolean/Phrase | Interface - EBSCOhost  Search Screen - Advanced Search  Database - CINAHL | 1,235 |
| S12 | TI neurastheni* OR AB neurastheni* | Search modes - Boolean/Phrase | Interface - EBSCOhost  Search Screen - Advanced Search  Database - CINAHL | 28 |
| S11 | TI ( (low or lack) n7 energy ) OR AB ( (low or lack) n7 energy ) | Search modes - Boolean/Phrase | Interface - EBSCOhost  Search Screen - Advanced Search  Database - CINAHL | 1,349 |
| S10 | TI ( fatigue* or astheni* or tiredness or tire or tired or weary or letharg* or apath* or malaise* ) OR AB ( fatigue* or astheni* or tiredness or tire or tired or weary or letharg* or apath* or malaise* ) | Search modes - Boolean/Phrase | Interface - EBSCOhost  Search Screen - Advanced Search  Database - CINAHL | 14,953 |
| S9 | (MH "Asthenia") | Search modes - Boolean/Phrase | Interface - EBSCOhost  Search Screen - Advanced Search  Database - CINAHL | 70 |
| S8 | (MH "Fatigue+") | Search modes - Boolean/Phrase | Interface - EBSCOhost  Search Screen - Advanced Search  Database - CINAHL | 8,823 |
| S7 | S1 OR S2 OR S3 OR S4 OR S5 OR S6 | Search modes - Boolean/Phrase | Interface - EBSCOhost  Search Screen - Advanced Search  Database - CINAHL | 28,827 |
| S6 | TI ( ((brain* or cerebr* or intracerebr* or crani* or intracran* or head* or subarachnoid* or subdural* or epidural* or extradural*) n3 (haematoma* or hematoma* or hemorrhag* or haemorrhag* or pressur* or bleed*)) ) OR AB ( ((brain* or cerebr* or intracerebr* or crani* or intracran* or head* or subarachnoid* or subdural* or epidural* or extradural*) n3 (haematoma* or hematoma* or hemorrhag* or haemorrhag* or pressur* or bleed*)) ) | Search modes - Boolean/Phrase | Interface - EBSCOhost  Search Screen - Advanced Search  Database - CINAHL | 6,023 |
| S5 | TI ( ((head* or cerebr* or crani* or capitis* or brain* or forebrain* or skull* or hemispher* or intracran* or orbit*) n3 (injur* or trauma* or lesion* or damag* or wound* or destruction* or swell* or oedema* or edema* or fracture* or contusion* or commotion* or pressur*)) ) OR AB ( ((head* or cerebr* or crani* or capitis* or brain* or forebrain* or skull* or hemispher* or intracran* or orbit*) n3 (injur* or trauma* or lesion* or damag* or wound* or destruction* or swell* or oedema* or edema* or fracture* or contusion* or commotion* or pressur*)) ) | Search modes - Boolean/Phrase | Interface - EBSCOhost  Search Screen - Advanced Search  Database - CINAHL | 19,797 |
| S4 | TI concuss* OR AB concuss* | Search modes - Boolean/Phrase | Interface - EBSCOhost  Search Screen - Advanced Search  Database - CINAHL | 1,160 |
| S3 | TI ( TBI or TBIs or mTBI or mTBIs ) OR AB ( TBI or TBIs or mTBI or mTBIs ) | Search modes - Boolean/Phrase | Interface - EBSCOhost  Search Screen - Advanced Search  Database - CINAHL | 3,221 |
| S2 | (MH "Head Injuries") | Search modes - Boolean/Phrase | Interface - EBSCOhost  Search Screen - Advanced Search  Database - CINAHL | 3,894 |
| S1 | (MH "Brain Injuries+") | Search modes - Boolean/Phrase | Interface - EBSCOhost  Search Screen - Advanced Search  Database - CINAHL | 12,807 |

**Additional file 2:** Characteristics of Excluded Studies

| N | Author, Date, Country | Reasons for exclusions | Citations |
| --- | --- | --- | --- |
|  |  |  |  |
|  |  |  |  |

**Additional file 3:** Quality assessment of studies using guidelines developed by Hayden et al, 2006

|  | Study | Study participation | Study attrition | Prognostic factor | Outcome | Confounding measurement and account | Analysis | Overall  assessment  of the study |
| --- | --- | --- | --- | --- | --- | --- | --- | --- |
