# Supplementary material for: Fatigue in adults with traumatic brain injury: predictors and consequences. A systematic review of longitudinal study protocols
Source: Syst Rev. 2013 Jul 11;2:57. doi: 10.1186/2046-4053-2-57 (PMC3717139; doi:10.1186/2046-4053-2-57)
Supplement: Additional file 2 — Characteristics of excluded studies. This file has a table of excluded studies. [file 2046-4053-2-57-S2.docx]

**Additional file 2:** Characteristics of Excluded Studies

| N | Author, Date, Country | Reasons for exclusions | Citations |
| --- | --- | --- | --- |
|  |  |  |  |
|  |  |  |  |
